# Considerations on amino acid patterns in the natural felid diet: a review

**DOI:** 10.3389/fvets.2024.1393890

**Published:** 2024-11-14

**Authors:** Mengmeng Sun, Annelies De Cuyper, Ellen S. Dierenfeld, Geert P. J. Janssens

**Affiliations:** Department of Veterinary and Biosciences, Faculty of Veterinary Medicine, Ghent University, Merelbeke, Belgium

**Keywords:** amino acids, felid, whole prey, tissues, muscle

## Abstract

Amino acids are essential for the growth, development, and reproduction of carnivores. This literature review summarizes the amino acid patterns of different raw diets including whole prey, body tissue and muscle for felids under human care. In general, natural prey (and its parts) meet the minimum essential amino acid requirements outlined by the National Research Council for adult cats. On a whole-prey diet, lysine and methionine far exceed requirements, while histidine approaches the minimum threshold. However, histidine concentration is higher in muscle meat. Body tissues, except for the skin, demonstrate no deficiency in essential amino acids. Notably, non-essential amino acids are found in raw meat diets in elevated concentrations, and their levels remain stable, akin to those of essential amino acids. Although felid requirements for non-essential amino acids are not specified, attention should be paid to their role in nutrition. While the amino acid patterns of diverse raw diets show no significant variation, the impact of prolonged single-source protein may require attention.

## 1 Introduction

Raw feeding has become a prevalent practice in the dietary regimen of carnivores in zoo settings. A survey of 44 zoos in Europe found that 98% chose to feed carnivores meat on the bone, and 95% chose to feed whole carcasses, but mainly small animals (rodents, rabbits, chickens) (1). Parts of the carcass still with fur or feathers (57%), whole meat (55%) and offal (45%) were used less frequently. At the same time, the trend of feeding domestic cats non-processed foods featuring alternative, diverse, or whole prey protein sources is gaining popularity (2). However, the specific impact of selecting different raw foods on amino acid (AA) intake remains unclear, warranting exploration to optimize dietary AA patterns.

Based on growth or nitrogen balance, AA can be divided into dietarily essential amino acid (EAA) or non-essential amino acid (NEAA) (3). Cats do not synthesize the carbon skeletons of twelve proteinogenic AAs: arginine (Arg), cysteine (Cys), histidine (His), isoleucine (Ile), leucine (Leu), lysine (Lys), methionine (Met), phenylalanine (Phe), threonine (Thr), tryptophan (Trp), tyrosine (Tyr), and valine (Val). These AAs are thus EAA for cats. Two of these AAs, Cys and Tyr become essential only in the absence of Met or Phe and are therefore considered “conditionally essential” (4). NEAA are eight AAs that cats can synthesize: alanine (Ala), asparagine (Asn), aspartic acid (Asp), glutamine (Gln), glycine (Gly), glutamic acid (Glu), proline (Pro), and serine (Ser) (5). Separate from growth, the maintenance AA requirements in adult felid stem from various physiological functions within the body. These requirements encompass the necessity to replenish AAs lost in areas such as the gastrointestinal tract, integument, and epidermis (6).

The aim of this study was to analyze the differences in AA patterns between whole prey, body tissue, and muscle meat. This analysis aimed to identify AA that may be overlooked in captive environments. Furthermore, a detailed examination of the AA patterns of individual tissues aimed to reveal which tissues play an important role in potential deviations from the ideal AA profile. Increased knowledge of individual behavioral selectivity toward specific carcass parts could help fine-tune the supply of AAs to achieve dietary nutritional balance.

## 2 Result and discussion

### 2.1 Amino acid recommendations

Table 1 compares recommended protein and EAA requirements for adult and growing domestic cats from multiple sources. In the NRC, nitrogen balance stands as the preferred dependent variable for establishing nitrogen and AA requirements across various life stages (7). Studies utilizing free AA diets and diets containing purified proteins revealed that the minimum crude protein requirement is about 18 g per 100 g dry matter (DM) for growing kittens (8, 9). Another investigation reported a calculated minimum daily protein intake for adult cats at around 16 g per 100 g DM (10). However, the discerning nature of adult cats, especially regarding the texture and taste of their food, poses challenges in accepting purified diets. Consequently, the dietary concentrations of AAs in some studies were calculated based on the EAA requirements of growing kittens. This methodology implies that the requirement for adult cats is mathematically derived and may overlook AA requirements for health and longevity. The AAFCO Feline Nutrition Expert Subcommittee (FNES) (11) made modifications to concentrations for some EAA to bring the recommended concentrations in line with the recommended allowance in the 2006 NRC and the FEDIAF Guidelines. FEDIAF protein levels are based on NRC (7) but adjusted for an apparent crude protein digestibility of 80% and the cat's energy intake (12).

**Table 1:** Protein and essential amino acid concentrations recommended for adult cats and growth kittens (maintenance and/or minimal requirement), g per 100 g dry matter (DM).

	**Protein**	**Arg**	**His**	**Ile**	**Leu**	**Lys**	**Met**	**Met+Cys**	**Phe**	**Phe+Tyr**	**Thr**	**Trp**	**Val**
**Adult cats**													
Minimum Requirement: AAFCO (11)	26.0	1.04	0.31	0.52	1.24	0.83	0.20	0.40	0.42	1.53	0.73	0.16	0.62
Minimum Requirement: FEDIAF (12)	25.0	1.00	0.26	0.43	1.02	0.34	0.17	0.34	0.40	1.53	0.52	0.13	0.51
Minimal Requirement: NRC (7)^a^	16.0	0.77	0.26	0.43	1.02	0.27	0.14	0.27	0.40	1.53	0.52	0.13	0.51
Maintenance: NRC (7)^b^	20	0.77	0.26	0.43	1.02	0.34	0.17	0.34	0.40	1.53	0.52	0.13	0.51
**Growth kittens**													
Minimum Requirement: AAFCO (11)	30.0	1.24	0.33	0.56	1.28	1.20	0.62	1.10	0.52	1.92	0.73	0.25	0.64
Minimum Requirement: FEDIAF (12)	28.0/30.0	1.07/1.11	0.33	0.54	1.28	0.85	0.44	0.88	0.50	1.91	0.65	0.16	0.64
Minimum Requirement: NRC (7)^a^	18.0	0.77	0.26	0.43	1.02	0.68	0.35	0.70	0.40	1.53	0.52	0.13	0.51
Recommended Allowance: NRC (7)^b^	22.5	0.96	0.33	0.54	1.28	0.85	0.44	0.88	0.50	1.91	0.65	0.16	0.64

The values for g/100 g DM have been calculated assuming a dietary energy density of 4,000 kcal ME/kg.

AAFCO, Association of American Feed Control Officials; FEDIAF, European Economic and Social Committee; NRC, National Research Council.

^a^The value comes from NRC's Minimal Requirement for cat. For adult cats, except for Lys and Met + Cys, which have minimum requirements, all other amino acids are based on adequate intake as the minimum requirements.

^b^The value comes from NRC's Recommended Allowance for cat.

In addition, the need to use protein as an energy source is not considered when taking nitrogen balance as a reference for requirement. Therefore, AA requirements of adult felids in real life may still deviate from the estimations made on the basis of growing kittens. Pezzali et al. used the indicator AA oxidation (IAAO) technology to determine the Met requirement for adult cats in the presence of excess cysteine (0.24% of DM), which is higher than current recommendations from the NRC, AAFCO, and FEDIAF (13). There is a growing need for AA requirement estimates for cats to be based on criteria such as optimal health, longevity, and optimum expression of coat color instead of criteria such as maximum growth, zero nitrogen balance, or minimum AA oxidation.

### 2.2 Amino acid patterns of whole prey

**Figure 1:**
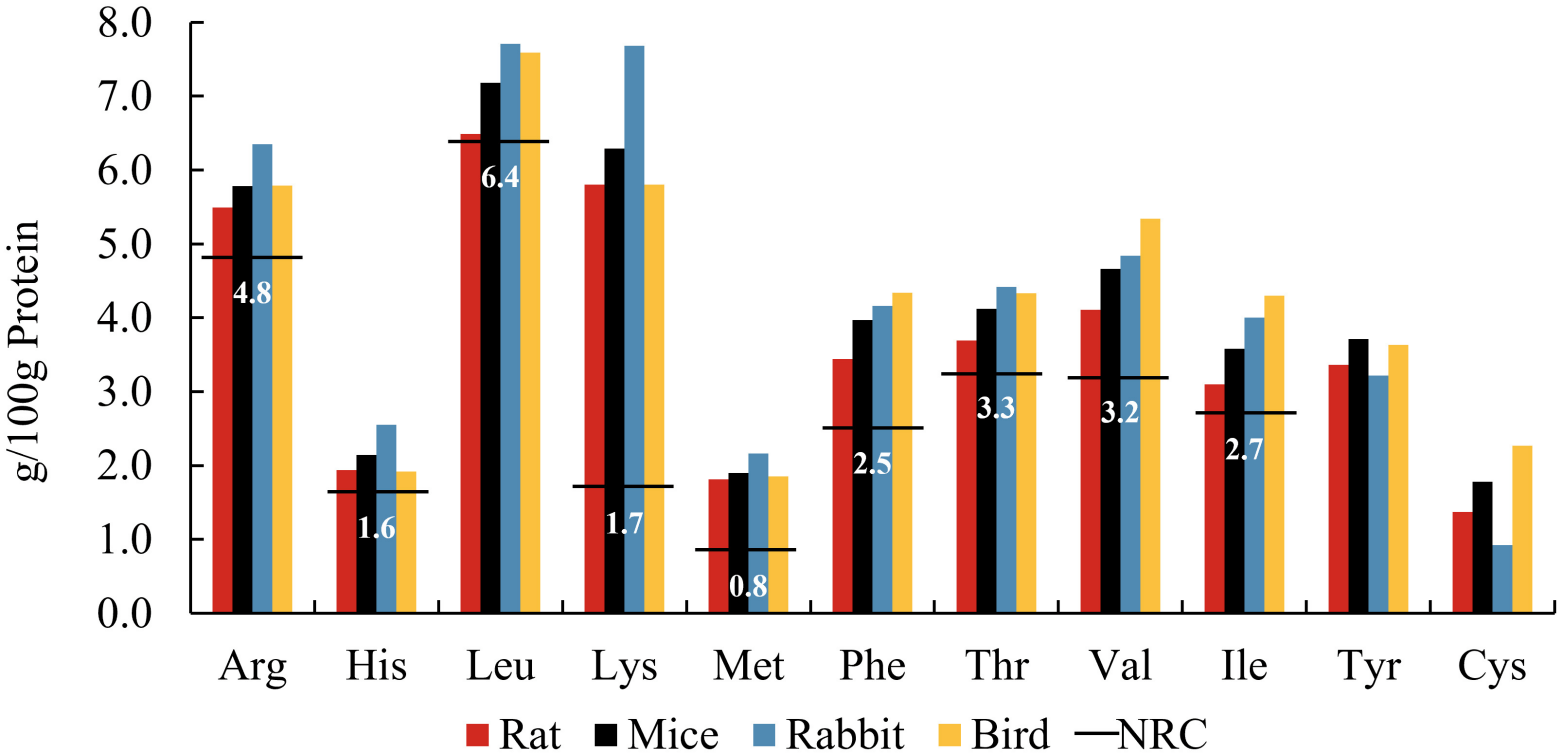
Amino acid patterns of whole prey including rats^a^, mice^a^, rabbits^b^, and birds^a^, compared to the NRC^c^ minimum requirements (horizontal lines) for adult cats. ^a^From (18). ^b^From (19). ^c^From NRC Minimal Requirement for adult cat (7).

The diet of wild cats often consists of entire small mammals, birds, and reptiles. One investigation documented that the diet of Hungarian wild cats comprised 50% voles and 20% mice, with other small mammals and birds making up the remaining 30% (14). Surveys of wild cat diets in France, New Zealand and the Canary Islands showed similar results (15–17). Figure 1 shows the AA patterns of whole carcasses extracted from smaller prey species (18, 19). The comprehensive analysis of whole prey underscores the adequacy of EAA meeting or exceeding the minimum requirements established by the NRC for adult cats (7). Some of these AAs far exceed requirements, such as Lys and Met. Lys, often a limiting AA in commercial cat foods due to lower concentrations in grains and susceptibility to Maillard reaction products (20, 21), is abundantly present in raw animal-based foods, effectively mitigating this constraint. There are no reported instances of clinical signs indicating acute or chronic toxicity associated with elevated levels of free Lys in felid diets. Met, on the other hand, is frequently the most limiting AA in cat diets formulated with natural ingredients (7). Given its role as a methyl donor essential for methyl transfer and protein-bound AAs, Met deficiency can lead to various metabolic abnormalities (22). It is crucial to meet the Met requirement, especially considering that Cys, another EAA, is synthesized exclusively from Met and cannot be converted back into Met (22). It was mentioned previously that recent research challenges the established minimum requirement for Met in adult cat diets (13). Nevertheless, the Met content in all small prey meets or exceeds these revised requirements, ensuring a minimum Met intake for felid health, yet without the risk of real excess, which could reduce lifespan (23). The diet of zoo felines often consists of bled carcasses. Blood is rich in AA, such as Lys (24). Therefore, the AA composition of blood should be carefully considered when designing a diet for zoo felines to better simulate the nutritional composition of the wild diet.

Amino acid patterns are relatively stable among species. However, the total amount of AAs provided in the diet may not entirely represent the amount fully available to cats, and its bioavailability is mainly affected by digestibility (25). Kerr et al. compared the AA digestibility of six commercially available prey species, including mouse, rat, rabbit, chicken, duck, and quail, suggesting that AA digestibility in whole carcasses varies by species (26). The coefficients of total EAA digestibility ranged from 84 to 94%. Within whole prey, a portion of the total protein derives from the skin, fur, and (raw) bones (typically referred to as animal fiber) (27), and these less digestible proteins may reduce the bioavailability of AAs (28). Although animal fiber may supply few bioavailable AAs via enzymatic digestion in the upper gut, these undigested nitrogenous compounds can enter the large intestine, where colonic microorganisms break them down, releasing peptides and AAs and producing bacterial metabolites, including ammonia, short chain fatty acid (SCFA), and branched-chain fatty acid (BCFA) (29). Preferred AA substrates for colonic bacteria include Lys, Arg, Gly, and the branched-chain AAs (Leu, Val, and Ile) (30). Microbial metabolism of AAs also produces biogenic amines. Biogenic amines are produced by the decarboxylation of AAs, such as cadaverine and putrescine from the decarboxylation of Lys and Arg, respectively (31). High concentrations of biogenic amines are generally considered to be detrimental to health (32). Whether negative or positive fermentation metabolites gain the upper hand in the hindgut most likely depends on the balance of fermentable and unfermentable animal fiber (33). For instance, fecal concentrations of most biogenic amines were lower in domestic cats fed whole mice compared with those fed extruded cat food, with the exception of agmatine and tyramine (34). It was therefore suggested that the more unfermentable fraction of animal fiber such as hair and skin may act as a bulking agent in the hindgut, mitigating the negative effects of AA fermentation.

### 2.3 Amino acid patterns of body tissues

**Figure 2:**
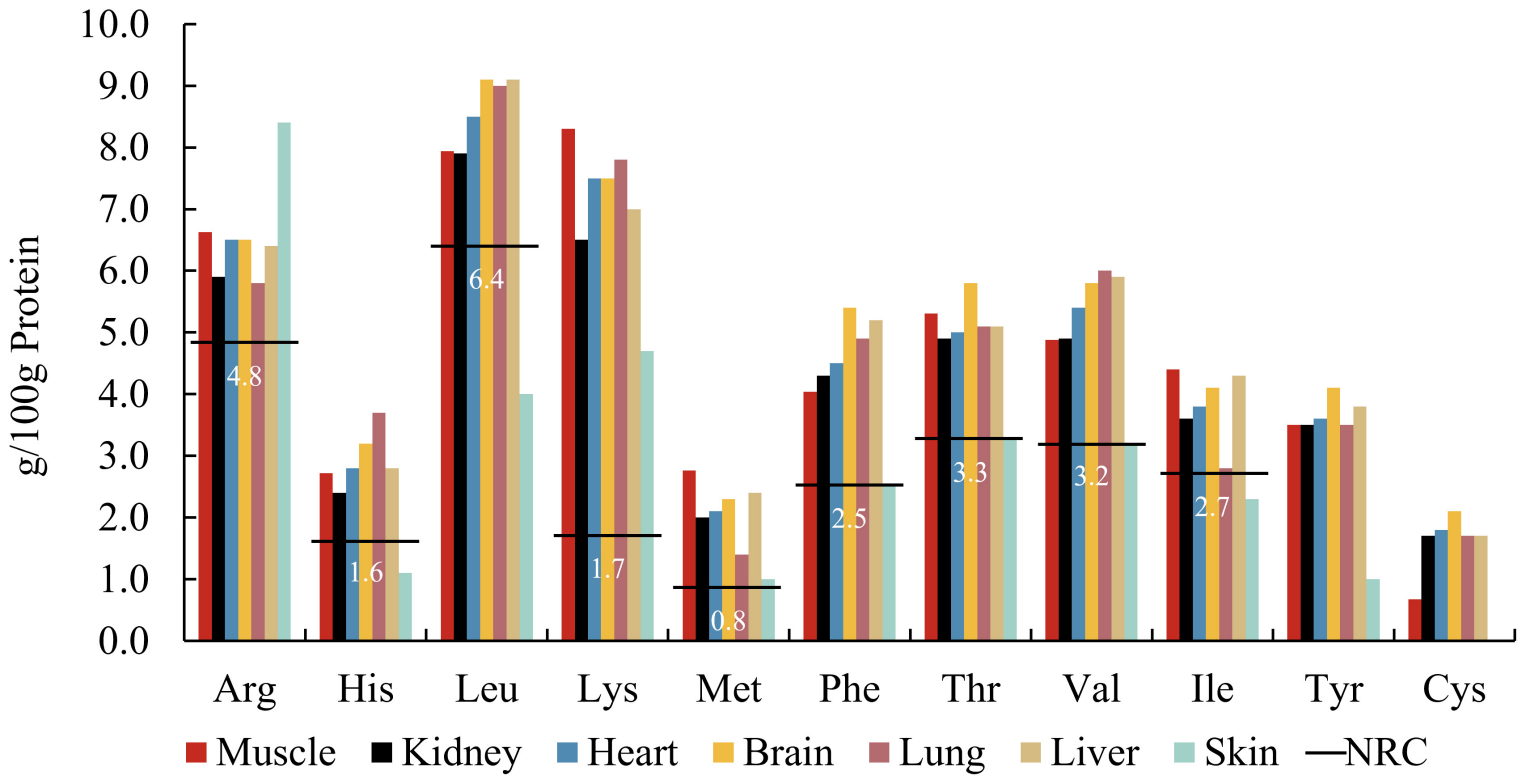
Amino acid patterns of rabbit tissues^a^ including muscle, kidney, heart, brain, lung, liver, and rabbit skin^b^, compared to the NRC^c^ minimum requirements (horizontal lines) for adult cats. ^a^From (19). ^b^From (38). ^c^From NRC's Minimal Requirement for adult cat (7).

Carnivore body size determines selection for specific prey sizes (6, 35, 36). Some large carnivores such as wolves, after successfully hunting prey, first share the entrails with pack mates and consume highly nutritious organs, then major muscle tissue, and finally bones and hides (37). Our analysis used rabbit tissues as a model for examining AA patterns across tissues (Figure 2) (19, 38). With the exception of skin, AA in all body tissues exceeded the NRC's minimum requirements. Current published data mainly comprises a selection of soft tissues, such as liver, lung, and kidney, with a lack of data on the AA composition of connective tissues containing collagen, such as skin, and hair. Studies on the AA composition of human tissues show that skin contained lower amounts of AAs, except for Gly, than liver, heart, and muscle (39). After comparing the AA composition of hair and epidermis (40), it was found that Cys is abundant in hair keratin, but it is rare in epidermal keratin. Tyr and its precursor Phe are most abundant in epidermal keratin whereas Tyr is less common in hair keratin. Therefore, feeding selectively on particular tissues may result in deviating AA intakes by carnivores, considering the AA composition of their natural diet. However, further research is needed on the AA digestibility of different tissues, especially whether the animal fiber components will affect the digestibility of AAs.

**Figure 3:**
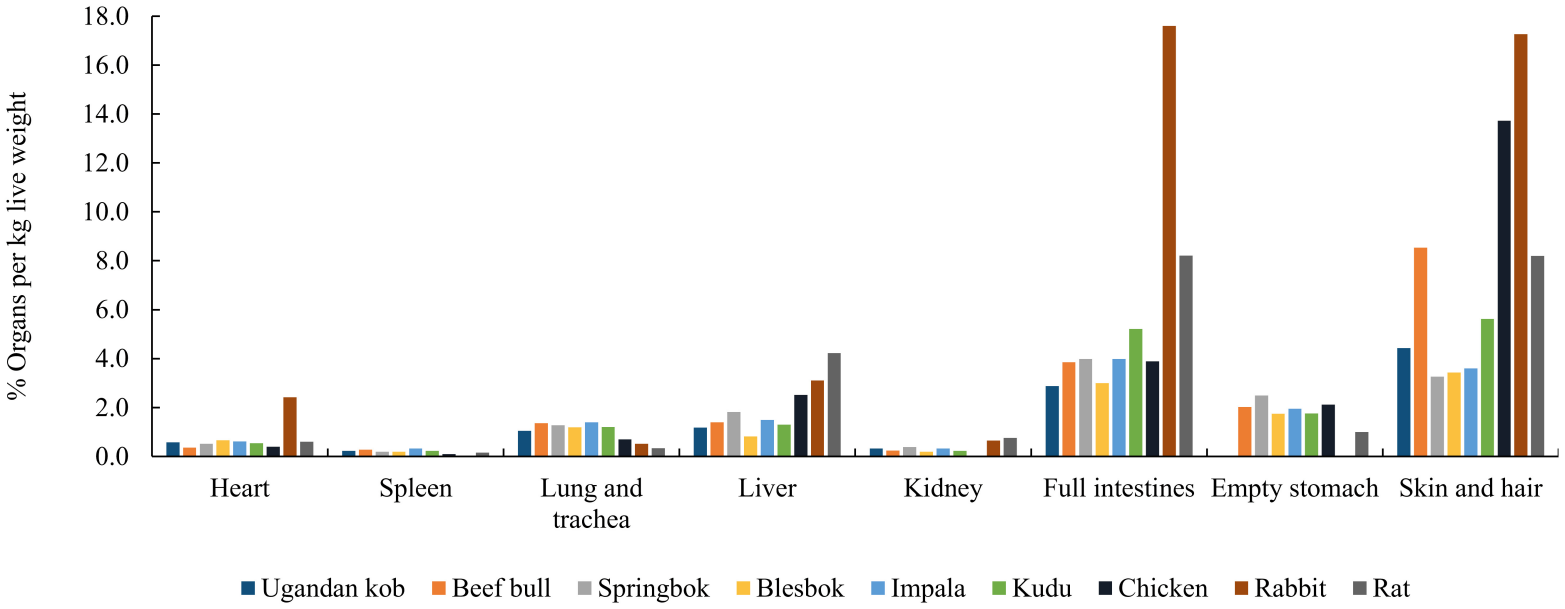
The % organ per kg live weight of Ugandan kob^a^, beef bull^b, c^, springbok^d^, blesbok^d^, impala^d^, kudu^e^, chicken^f^, rabbit^g^, rat^h^. ^a^Mean values from male and female Uganda kob calculated from (42). ^b^ From (43); ^c^ Calculated from (44) by dividing the slaughter weight with the respective organ weights to obtain the % of organ per kg dead bull weight. ^c^From Chala Merera Erge. ^d^Mean values for male and female calculated from (45) by multiplying the proportional distribution % of each organ or carcass item with the dressing % and dividing it by 100. ^e^From (46). ^f^From (47). ^g^From (48). ^h^From (49).

Compared to the body tissues of small prey, the study by Beach et al. (41) highlighted AA variations among bovine organs, implying that the way a carnivore feeds on prey may affect its dietary AA pattern. We compared and summarized the proportions of different organs and carcass parts in different animals [Uganda kob (42), beef bull (43, 44), springbok, blesbok, impala (45), kudu (46), chicken (47), rabbit (48), and rat (49), respectively] (Figure 3). These data demonstrated that the proportional percentages per live weight of heart, spleen, lungs and trachea, liver, kidney, full intestines, empty stomach, and skin and hair are roughly the same for most of these medium-sized bovid species. However, some small prey such as chickens, rabbits, and rats, contain different proportions of organs compared with larger prey. Although values were summarized from different studies and from different publication years, this difference in % organ per kilogram live weight among species may have a critical effect on the AA consumption of carnivores eating different species of prey.

### 2.4 Amino acid patterns of muscle meat

**Figure 4:**
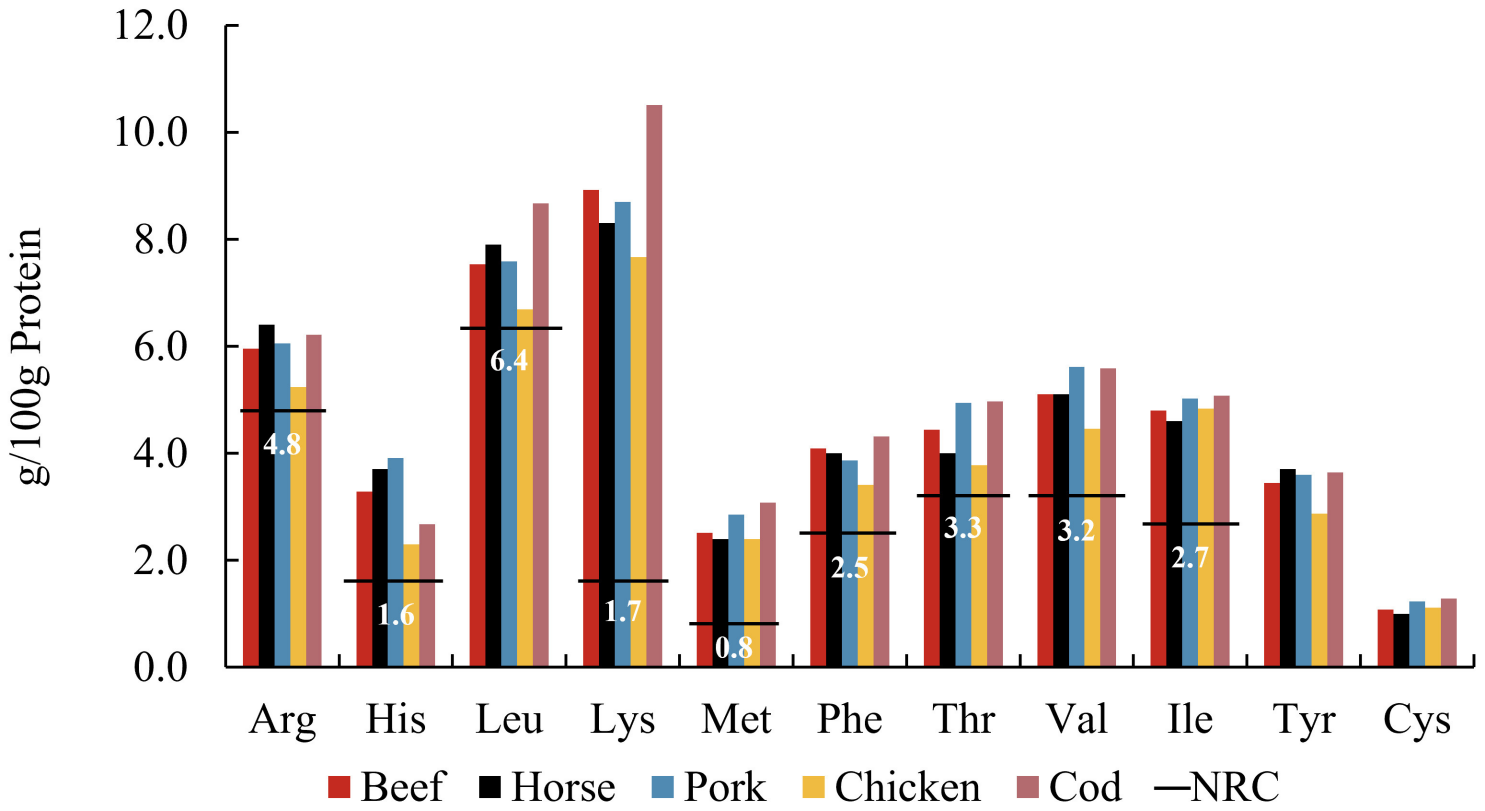
Amino acid patterns of muscle meat^a^ including beef, horse, pork, chicken, and cod, compared to the NRC^b^ minimum requirements (horizontal lines) for adult cats. ^a^From FAO Nutritional Studies (79). ^b^From NRC's Minimal Requirement for adult cat (7).

Optimal feeding strategies for zoo carnivores remain a subject of considerable interest, with nutritional recommendations for domestic cats (50) serving as a reference framework in managing diets for all carnivores under human care. The use of commercially prepared raw-meat diets is a well-known and frequently used diet type in zoological institutions globally (51). In zoos, due to logistic and financial constraints, muscle meats are often the main animal-derived dietary component, routinely rotated among prey species to provide dietary diversity. The AA patterns of different muscle meats are compared in Figure 4. Common raw meats were purchased from retailers and included beef, horse, pork, chicken breast, and cod. The individual AA content of chicken breast is slightly lower than that of other muscles, while cod was relatively higher. Overall, however, the AA pattern was relatively stable across muscle meats of widely varying potential prey species. This implies that the AA pattern in muscle proteins appears similar across the animal kingdom, which would allow a consistent supply of AAs regardless of prey species, if diet were predominantly flesh.

Meat protein generally contains a high amount of crude protein and is considered easily digestible by enzymes (52). It is not known if long-term feeding of solely muscle meat as a major protein source to carnivores may affect their health. However, human studies suggest that a high-protein diet may increase the risk of chronic kidney disease (53), with daily consumption of red meat posing a higher risk (54). Additionally, high-protein diets are associated with various metabolic complications, potentially impairing kidney health (55). A study in dogs found that high protein intake increases glomerular filtration (56), and hyperfiltration is considered an early marker of kidney damage in prediabetes and prehypertension (57). Traditionally, cats, as obligate carnivores, are believed to have higher dietary protein requirements than omnivorous mammals. However, Eisert reconsidered the protein requirements of cats, proposing a hypothetical model in which cats and other small hypercarnivores do not have high protein requirements *per se*, but rather have high endogenous glucose requirements which are met through gluconeogenesis based on EAA (58). We postulate that the composition of whole prey, compared to a muscle-only diet, may offer a more balanced nutritional profile. Whole prey not only provides the necessary AAs for gluconeogenesis, fulfilling the glucose needs of the brain and other organs but also offers animal fiber that supports beneficial gut fermentation, promoting overall health.

### 2.5 Elaborating some cases showing the potential impact of NEAA

**Figure 5:**
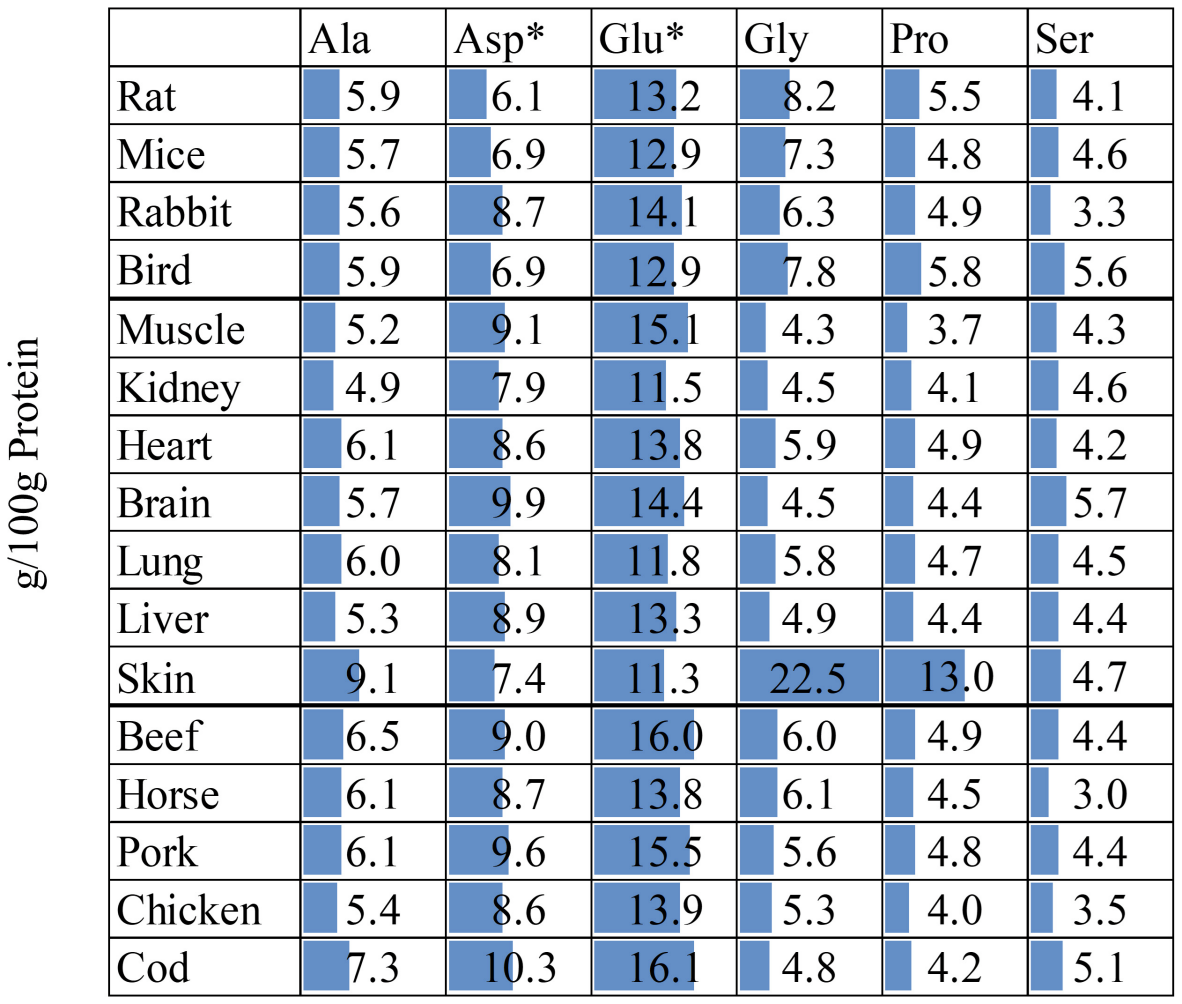
Non-essential amino acid content (g/100g protein) in whole prey^a^, body tissue^b,c^, and muscle meat^d^. *Asp is the sum of Asp + Asn; Glu is the sum of Glu + Gln. Deamidation during sample preparation which converts Asn to Asp and Gln to Glu. ^a^From (18); rabbit date from (19). ^b^From (19); rabbit skin data from (38). ^c^From (38). ^d^From FAO Nutritional Studies (79).

Most protein research has focused on the dietary composition of EAA. In contrast, ingredients of diets containing NEAA that can be completely formed in the body have received little attention (59–61). Thus, we further compared the NEAA content in whole prey, body tissues and muscle meat (Figure 5). Studies in pigs, poultry, and fish provide increasing evidence that animals indeed have dietary requirements for NEAA (62) that appear critical for achieving animal genetic potential in terms of growth, reproduction, lactation, production performance, and overall health. This prompts a reconsideration of felid needs in the context of NEAA. Kittens fed a diet containing 35% protein comprising solely EAA, but without any NEAA, grew at a suboptimal rate, compared with feeding a diet containing 25% crude protein containing both EAA and NEAA (63). Examples of NEAAs that are increasingly studied for their roles in metabolism. Dietary Gly supplementation can partially mitigate oxidative stress-related glutathione deficiency in elderly cats by increasing initial erythrocyte glutathione levels and modifying oxidative stress markers (64). In mammals including dogs, dietary Asp, Glu, and Gln, along with arterial Gln, serve as primary metabolic fuels in the small intestine (65). Carnivorous fish, such as the hybrid striped bass, rely on Glu and Gln as primary metabolic fuels in various organs, including the proximal intestine, liver, kidneys, and skeletal muscle (66). Furthermore, the critical dependence of gut function on the provision of dietary Gln is observed under various gastrointestinal disorders (62), for example captive cheetahs often experience progressive gastritis, while wild cheetahs infected with the same *H. pylori* strain do not develop this condition. This difference may be attributed to variations in their immune responses (67). However, studies suggest that dietary Gln supplementation can alleviate the symptoms of *H. pylori* infection, reduce stomach inflammation, and improve overall gastrointestinal health. Adding 5% L-Gln to the diet of mice infected with *H. pylori* significantly reduced inflammation and lobular tissue proliferation (68). *Helicobacter suis* infected animals supplemented with Gln showed smaller gastric lymphoid follicles and less diffuse lymphoid infiltration compared to the animals receiving the standard diet (69). This protective effect might be amplified with higher levels of dietary Gln supplementation. This potential benefit extends to cats, particularly those dealing with conditions like cancer and intestinal damage (70). Pro plays an important role in protein synthesis, metabolism, nutrition, wound healing, antioxidant response, and immune response (71). Arg, Glu, and Gln (all NEAA) are potential substrates for the synthesis of Pro (72). Most mammals (including pigs, cattle, and sheep) can use Gln and Glu to synthesize Pro in the small intestine through the pyrroline-5-carboxylate (P5C) synthetase pathway (73). In carnivores (such as cats and ferrets), intestinal cells and other cell types lack P5C synthetase, so this pathway cannot be used for Pro synthesis (72), leaving Arg as the only precursor substrate in carnivores. However, Arg is an essential AA for cats (7) with a high demand used to maintain an active liver urea cycle and nitrogen balance throughout the body (74). Feeding cats and kittens an Arg-poor diet results in symptoms of hyperammonemia (e.g., salivation, emesis, neurological abnormalities, ataxia, tetany, and coma) within hours, and could progress to cyanosis and death due to respiratory failure (75–77). Dietary supplementation of Pro can compensate for some of the Arg in these animals.

As with EAA, the content of various NEAA was relatively stable in different tissue types, except for skin. The main component of skin is collagen and is therefore rich in Gly and Pro (78). An *in vitro* digestion experiment of different tissues in rats showed that the digestibility of Gly and Pro in skin with fur was 81.0 and 69.5%, respectively, and correlation analysis showed that their digestibility and concentration were negatively correlated (28). Although these AAs account for a large proportion of skin/hair and bone, their impact on bioavailable AA intake is limited. Therefore, when only muscle or skinned carcasses are fed, the impact of these AAs will be low. However, if animals are encouraged to consume additional less digestible carcass components such as skin, cartilage and bone, this may still have limited effects on bioavailable AA intake, however, may have other beneficial physiological effects on carnivores.

## 3 Conclusion

When studying feline nutrition, AA composition is important but not the only factor to consider. Beyond the AA requirements and bioavailability of (adult) obligate carnivores, other nutrients and diet traits are important to study.
